# A Rare Presentation of *In Situ* Mantle Cell Lymphoma and Follicular Lymphoma: A Case Report and Review of the Literature

**DOI:** 10.1155/2014/145129

**Published:** 2014-11-16

**Authors:** Josephine Taverna, Anju Nair, Seongseok Yun, Spencer Paulson, Jonathan H. Schatz, Daniel Persky, Deborah Fuchs, Soham Puvvada

**Affiliations:** ^1^Division of Hematology and Oncology, Department of Medicine, University of Arizona Cancer Center, University of Arizona, USA; ^2^Department of Medicine, University of Arizona, USA; ^3^Department of Pathology, University of Arizona, USA

## Abstract

A 65-year-old gentleman presented with left groin swelling over the course of two months. Physical exam revealed nontender left inguinal adenopathy, and computed tomography scans detected multiple lymph nodes in the mesenteric, aortocaval, and right common iliac regions. An excisional lymph node biopsy was performed. Pathologic evaluation demonstrated follicular center site which stained positive for PAX5, CD20, CD10, Bcl-2, Bcl-6, and mantle zone cells. These findings demonstrated CCND1 and CD5 positivity, suggesting composite lymphoma comprising follicular lymphoma (FL) with *in situ* mantle cell lymphoma (MCLIS). FL is known as indolent non-Hodgkin lymphoma; however, the clinical significance of a coexisting MCLIS continues to be elusive, and optimal management of these patients remains largely unknown. This case illustrates the diagnostic and therapeutic challenges of composite lymphomas. This paper also discusses advances in molecular pathogenesis and lymphoma genomics which offer novel insights into these rare diseases.

## 1. Introduction

Composite lymphoma (CL) is defined by two or more morphologically and immunophenotypically distinct lymphomas observed within the same anatomic site [1]. Only 6 cases of composite FL and MCL lymphoma have been reported to date (Table 1) [2–7], and FL with coexistent MCLIS cases have only been described in 6 patients (Table 2) [5–9]. The FL component typically appears to be low grade, harbors the t(14; 18) translocation, and stains positive for Bcl-2 on immunohistochemistry (IHC). The MCL component, however, displays a diffuse or* in situ* mantle-zone growth pattern, harbors the t(11; 14) translocation, and expresses CCND1 [10, 11]. The incidence of CL ranges from 1–4.7% of total lymphomas and has no gender or ethnic predilection. Here, we add one more case to the rare collection of FL with “*in situ*” MCL, where such findings are often incidental and associated with indolent clinical course.

## 2. Case Presentation

**Figure 1:**
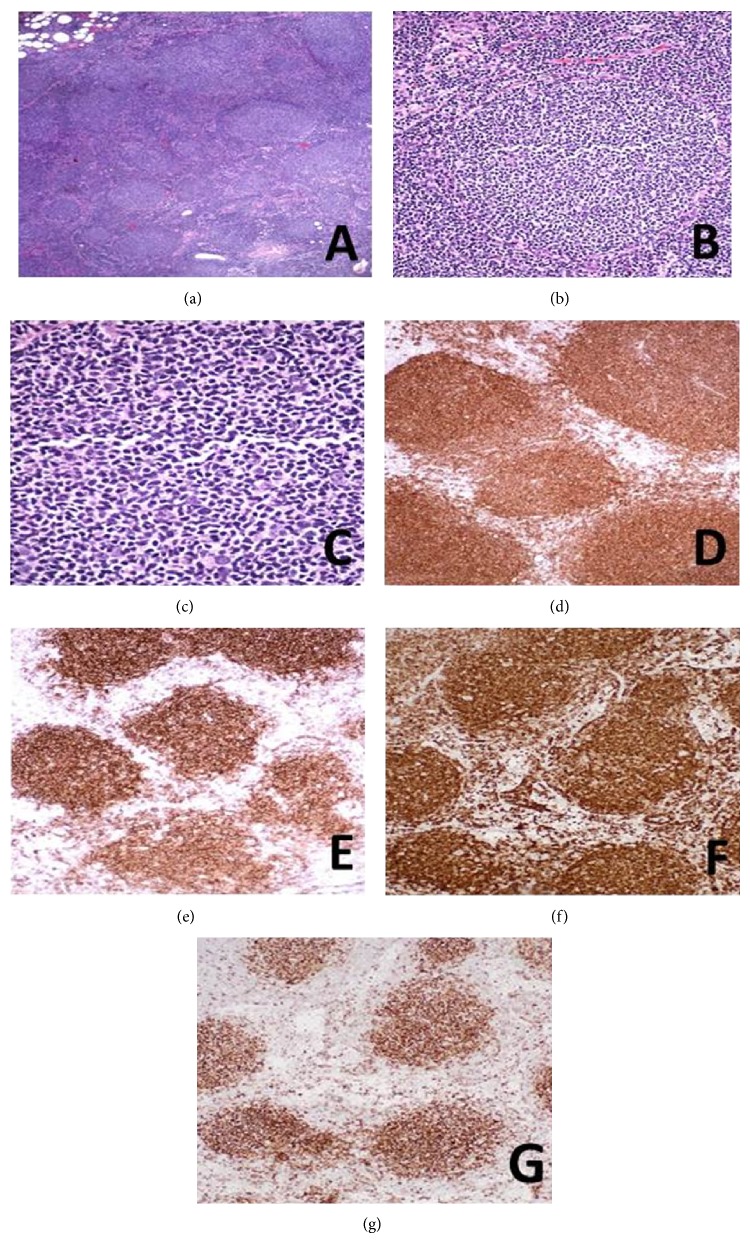
H & E and immunohistochemical staining of follicular components. Hematoxylin and eosin stained sections showed numerous neoplastic follicles occupying almost the entire lymph node, effacing the normal nodal architecture extending from the cortex to the hilum, and invading beyond the capsule ((a) 4x). The neoplastic follicles consist of small centrocytes with ovoid shape, small angulated nuclei, clumped chromatin, and inconspicuous or absent nucleoli. Rare intermixed centroblasts are seen ((b) 20x and (c) 40 xs). Immunohistochemical staining revealed the germinal center cells expressing the pan B-cell marker CD20 ((d) 10x) in addition to germinal center-associated markers CD10 ((e) 10x), BCL-6 ((f) 10x), and BCL-2 ((g) 10x). The collective histologic and immunophenotype findings indicated a follicular lymphoma, grade 1 of 3.

**Figure 2:**
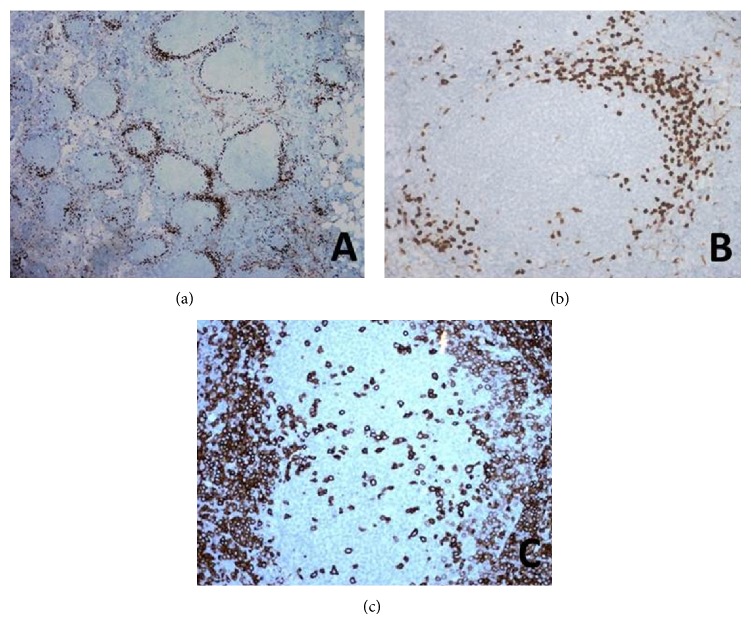
Immunohistochemical staining of* in situ* mantle cell lymphoma components. Cyclin D1 immunohistochemistry revealed a peripheral rim of positive cells within the mantle zones of the majority of the neoplastic secondary follicles ((a) 4x and (b) 20x). These cells appeared to show dim coexpression of CD5 ((c) 20x).

A 65-year-old male with a history of gastric bypass surgery and left inguinal hernia repair presented with left groin swelling for two months without any other associated symptoms. He initially presented with an enlarged, palpable, nontender left inguinal lymph node measuring 1 × 1.5 cm. Initial laboratory results, including complete blood count, complete metabolic panel, lactate dehydrogenase, and beta-2-microglobulin, were normal. Computed tomography scan detected multiple lymph nodes in the mesenteric, aortocaval, and right common iliac lymph nodes measuring up to 13 mm in diameter. At the time of surgical evaluation, the inguinal lymph node had regressed and was difficult to pinpoint by physical exam. He, therefore, underwent excisional biopsy of a mesenteric lymph node visible on the CT scan (Figure 1). The follicular center site stained positive for PAX5, CD20, CD10, Bcl-2, and B-cell lymphoma 6 (Bcl-6), indicating follicular lymphoma. However, CCND1 unexpectedly highlighted CD5 positive mantle zone cells in neoplastic follicles, which suggested a MCLIS (Figure 2) component. FISH analysis demonstrated 35% of t(11; 14) and 65% of t(14; 18) in tested nuclei that are mutually exclusive. Further workups including bone marrow biopsy and aspirate were unremarkable. Colonoscopy with random biopsies was negative with no evidence of malignancy although esophagogastroduodenoscopy was not feasible due to his recent gastric bypass surgery. The patient has been followed up for 2 years since initial diagnosis of composite lymphoma and is currently under active clinical surveillance without any signs of disease progression.

## 3. Discussion

FL is one of the common non-Hodgkin lymphomas with an estimated incidence of 3.18 cases per 100,000 people in the USA [19]. FL can present with asymptomatic lymphadenopathy and bone marrow involvement; however, extranodal involvement is relatively uncommon [20]. FL is the prototype for indolent lymphomas and has a median overall survival of greater than 10 years with current treatment paradigms [20]. FL arises from follicular germinal centers within lymph nodes, when naïve B-cells undergo somatic hypermutation (SHM) in the VH region of the immunoglobulin genes during antigen stimulation. Normally, B-cells with lower affinity to antigen undergo apoptosis (negative selection); however, the FL precursors with t(14; 18) (q32; q21) rearrangement are able to survive through negative selection due to constitutive Bcl-2 overexpression. During SHM, additional point mutations, deletions, and duplications are introduced in the DNA sequence of VH region that can contribute to pathogenesis of FL [21].

MCL is an aggressive and relatively rare lymphoma with an annual incidence of approximately 4–8 cases per million in the USA [22, 23]. 70% of MCL cases present with advanced disease characterized by splenomegaly, nodal, and/or extranodal involvements [24, 25]. A small subset of patients may follow an indolent clinical course, although clinical progression warrants early therapeutic intervention [25, 26]. Morphologically, five cytological variants of MCL have been recognized: classic, small cell, marginal zone-like, pleomorphic, and blastoid. The t(11; 14) (q13; 32) chromosomal rearrangement is the hallmark of MCL, and it results in overexpression of protooncogene, CCND1 [27].

*In situ* lymphomas are recognized in the WHO classification of both MCL and FL. They are usually incidental findings in reactive-appearing lymph nodes [28].* In situ* mantle cell lymphoma (MCLIS) is characterized by CCND1 positive MCL-like cells restricted to the mantle zone of hyperplastic follicles in reactive lymphoid tissues of healthy individuals [12]. MCLIS is extremely rare with only 22 cases reported to date. It has a heterogeneous clinical presentation with 6 cases demonstrating extranodal disease (i.e., lacrimal glands, nasopharynx, oropharynx, and gastrointestinal tract), 1 case with splenic involvement, and bone marrow involvement in 26% of reported cases (Table 3) [8, 12–18]. The diagnosis of MCLIS is often made incidentally from biopsies performed during lymphoma workup. Morphologically, the architecture of the lymphoid tissue remains intact and reactive follicles are mainly distributed in the cortical areas. The mantle zones of these follicles are preserved and CCND1 positive cells are often restricted to mantle zones [12].

Most of MCLIS cells can be divided into two groups: CD5-negative and CD5-positive MCLIS. CD5-negative MCLIS can be typically seen in younger patients, where it often presents with nodal involvement and requires no treatment in most cases. On the other hand, CD5-positive MCLIS is associated with older age, extranodal involvement, and other lymphomas. Patients with CD5-positive MCLIS are more likely to require treatment. Interestingly, no difference in survival has been noted between these two groups [15].

The t(11; 14) (q13; q32) rearrangement juxtaposing the protooncogene CCND1 to the immunoglobulin heavy chain (IGH) complex is considered a pivotal event in the development of MCL. This translocation occurs within the bone marrow during pre-B stage differentiation with V (D) J recombination of the IGH variable region (IGHV) [10]. It is hypothesized that the naive B-cell carrying t(11; 14) colonizes the mantle zone of the lymphoid follicle, generating an* in situ* MCL lesion. Two distinct populations of MCLIS tumors have been recently described. SOX11-positive MCLIS tumors reside in the mantle zone of lymphoid follicles, are genetically unstable, and undergo limited IGHV somatic mutations. Alternatively, SOX11-negative MCLIS tumors are genetically stable and arise from naïve B-cells harboring t(11; 14) which enter the germinal center and undergo* IGHV *somatic hypermutations. Carvajal-Cuencia et al. found SOX11 expression in 44% of 16 cases with MCLIS and hypothesized that MCLIS may represent an early step in MCL lymphomagenesis [12]. One would presuppose that SOX-positive MCLIS and FL are clonally unrelated and mutually exclusive. However, previous studies show that 15–40% of MCLs carry IGHV hypermutations with a strong bias in the IGHV gene repertoire [29]. Collectively, these results suggest that both lymphomas may originate from the same preneoplastic clone. Unfortunately, depletion of material from confirmatory pathological studies did not permit PCR evaluation for clonality in our patient.

No guidelines have been established for the staging and management of MCLIS. The majority of patients with MCLIS will not develop overt MCL. Therefore, they can be followed up for long periods without treatment [12]. Tumor surveillance with imaging only in the presence of disease-related symptoms or organ involvement appears reasonable. MLCIS must be distinguished from mantle cell lymphoma with a mantle zone pattern and overt mantle cell lymphoma because their treatment approaches vary. Accordingly, staging workup to exclude other sites of involvement or rule out the coexistence of an overt lymphoma is recommended. When FL and MCLIS are reported simultaneously, the dominant lymphoma should be treated when patient requires treatment [30]. Our patient does not meet any indications for treatment, and he has been under active clinical surveillance for 2 years without any signs of disease progression. If there is any evidence of disease progression, we plan to repeat complete staging work-up including a bone marrow and lymph node biopsy with PCR analysis.

## 4. Conclusion

The significance of MCLIS still remains obscure. At this moment, it is unknown whether MCLIS represents true precursor lesions that will progress to an overt lymphoma or are incidental findings with a low chance of progression. Composite lymphoma with FL and MCLIS can pose diagnostic and therapeutic challenges. However, with new advances in molecular pathology and lymphoma genomics, we have more opportunities to investigate these rare diseases and gain novel insights into their biology in order to benefit the management of affected patients.

## Figures and Tables

**Table 1:** MCL and FL composite lymphomas.

Case number	Age	Involvement	Immunohistochemistry staining	Treatment	Follow-up	Ref
	Gender					
1	66 F	Waldeyer ring Tonsil Pharyngeal wall Lingual tonsil Nasal cavity	MCL: CD5+, CD20+, IgD+, cyclinD1+, Bcl-2+, CD3− FL: CD2+, CD10+, Bcl-2+, Bcl-6+, CD5−	Corticosteroid	PET scan every 3–6 months	[2]

2	M	Ocular adnexa	NA	NA	Poor prognosis mentioned	[3]

3	84 F	Spleen	FL: CD20+, CD23+, Bcl-6+, CD5−, CD43−, Bcl-2− MCL: cyclinD1+, CD5−	Splenectomy Patient refused additional treatment	9 months after splenectomy, CT scan showed intra-abdominal lymphadenopathy and patient died from unknown cause 13 months later	[4]

4	70 F	Cervical LN Inguinal LN	CD20+, CD3−, cyclinD1− 50% of B-cell expressed CD5 (MCL) 30% of B-cell expressed CD10 (FL)	No chemotherapy	CT of chest and abdomen showed no evidence of lymphadenopathy or hepatosplenomegaly	[5]

5	65 M	Inguinal LN	FL: CD20+, CD79a+, CD10+, Bcl-2+, CD5−, CD230, cyclinD1−, p27+ MCL: CD20+, CD79a+, CD5+, CD10−, CD23−, cyclinD1+, p27− Interfollicular areas mostly CD5+ showing distinct cylinD1 staining	Splenectomy	MCL caused disease progression into spleen. One year after splenectomy, it achieved stable disease	[6]

6	58 F	Mesenteric LN Small bowel	MCL: CD5+, CD20+, CD43+ FL: CD10+, CD20+, p27+	22 cycles of chemotherapy over 2 years	Complete remission	[7]

**Table 2:** MCL *in situ* with FL.

Case number	Histology	Immunohistochemistry flow cytometry	FISH/southern blot/PCR	Tissue microdissection	Molecular technique to determine clonal relationships	Results interpretation	Ref
1	MCL: *In situ* FL: LG/G1	*In situ* MCL: CD5−; cyclin D1+; CD23− FL: CD10+; BCL2+	NA	Not done	Not done	Not known	[5]

2	MCL: *In situ* FL: LG/G1	*In situ* MCL: CD5+; cyclin D1+; CD23− FL: CD10+; BCL2+	t(11; 14) + t(14; 18) +	Performed	IgH PCR FR1 FR3 JH	Clonally related	[6]

3	MCL: *In situ* FL: LG/G1-2, *in situ *	*In situ* MCL: CD5−; cyclin D1+; CD23− FL: CD10+; BCL2− *In situ* FL: CD10+; BCL2+	t(11; 14) + t(14; 18)+	Performed	IgH PCR FR2 D1–6 IgL PCR Vk/Kde	Clonally related	[7]

4	MCL: *In situ* FL: Stage 4B	*In situ* MCL: CD5−; cyclin D1+; CD23− FL: CD10+; BCL2+	NA	Not done	Not Done	Not known	[8]

5	MCL: *In situ* FL: NA	*In situ* MCL: CD5+; cyclin D1−; CD23− FL: CD10+; BCL2+	t(14; 18)(q32; q21) + t(11; 14)(q13; 32) +	Performed	PCR for IgH	Clonally related	[9]

6	MCL: *In situ* FL: NA	*In situ* MCL: CD5+; cyclin D1+; CD23 NA FL: CD10+; BCL2+	t(11; 14) + t(14; 18) +	Performed	PCR for IgH	Clonally related	[9]

**Table 3:** Clinical features, follow-up, and management in *in situ* MCL lesions.

Case number	Age	Site of biopsy	Management	Follow-up	Status	CD5	Concurrent malignancy	Ref
	Sex							
1	70 M	Cervical lymph node	W&W	4 years	Overt MCL	−		[8]
2	65 F	LN	Chemotherapy	0.5 years	AND	−		[12]
3	65 M	Appendix	W&W	4 years	Overt MCL	+		[12]
4	66 M	Pelvic LN	W&W	4 years	Overt MCL	+	Prostate cancer	[12]
5	68 M	LN, mediastinal	W&W	1 year	AWD	Not tested		[12]
6	82 M	Oropharynx	W&W	3 years	AWD	+	CLL/SLL	[12]
7	82 M	Lymph node	Chemotherapy	1.5 years	AND	+	CLL/SLL	[12]
8	80 M	Inguinal LN	Chemotherapy	N/A	N/A	+	CLL/SLL	[12]
9	42 F	Cervical lymph node	W&W	1 year	Alive with no disease (AND)	−	Breast cancer	[12]
10	78 F	Lacrimal gland	Not available (NA)	NA	NA	+	NA	[12]
11	42 M	Supraclavicular LN	Radiotherapy	1.7 years	AND	−	Castleman disease	[12]
12	58 M	Intestine	Chemotherapy	1.4 years	AND	+	none	[12]
13	42 F	LN, axillary/inguinal, GIT	Chemotherapy	6 years	AND	+		[13]
14	70 F	LN, submandibular	W&W	12 years	AWD in Peripheral blood (PB)	+	Nonspecific granulomas	[14]
15	59 M	Cervical lymph node	W&W	5 years	AND	−	Papillary thyroid cancer	[15]
16	80 M	Cervical LN	Chemotherapy	1.5 years	Died	+	FL	[15]
17	65 F	Intramammary LN	Chemotherapy	5 years	AND	+	FL	[15]
18	65 M	Appendix	W&W	4 years	MCL	+	NA	[15]
19	68 M	Mediastinal LN	W&W	1 year	AND	NA	—	[15]
20	41 F	Inguinal lymph node (LN)	Watch and Wait (W&W)	19.5 years	Alive with disease (AWD)	−	none	[16]
21	72 F	Cervical lymph node	Radiotherapy	2 years	AND	+	Breast cancer	[17]
22	34 M	Left supraclavicular LN	Chemotherapy	1 month	Died	+	FL	[18]

W&W: watch and wait.

AND: alive with no disease.

AWD: alive with disease.

NA: nonavailable.

CLL: chronic lymphocytic leukemia.

SLL: small lymphocytic lymphoma.

LN: lymph node.

GIT: gastrointestinal tract.
